# Lipedema as a disorder of adipose tissue heterogeneity: insights from single-cell and spatial transcriptomics

**DOI:** 10.3389/fcell.2026.1809914

**Published:** 2026-06-17

**Authors:** Isabella Reid, Syeda Hafsa Ali, Ramin Shayan, Tara Karnezis

**Affiliations:** 1 O’Brien Institute Department, St. Vincent’s Institute of Medical Research, Melbourne, VIC, Australia; 2 Department of Medicine, St Vincent’s Hospital, University of Melbourne, Melbourne, VIC, Australia; 3 Department of Surgery, University of Melbourne, Melbourne, VIC, Australia; 4 Department of Plastic Surgery, The Alfred Hospital, Melbourne, VIC, Australia

**Keywords:** adipose, adipose depot, adipose stem cells, ECM, fat atlas, lipedema, single cell sequence (scRNA-seq), stem cells

## Abstract

Lipedema is a chronic adipose tissue disorder characterised by disproportionate accumulation of subcutaneous fat within specific anatomical depots, most commonly the lower extremities, with relative sparing of the trunk. Despite increasing clinical recognition, the mechanisms underlying the selective vulnerability of particular adipose depots and the progressive tissue remodelling observed in lipedema remain poorly understood. Emerging evidence suggests that lipedema is associated with complex alterations in adipocyte biology, adipose stem and progenitor cell (ASPC) function, immune signalling, vascular integrity, extracellular matrix remodelling, and lymphatic homeostasis, indicating that the disease extends beyond simple fat accumulation. Recent advances in adipose tissue biology have demonstrated that adipose depots are not functionally uniform structures, but rather anatomically distinct cellular ecosystems with unique developmental origins, transcriptional programs, stromal composition, immune niches, and metabolic properties. These depot-specific characteristics may provide an important framework for understanding the regional distribution and progression of lipedema. However, while substantial progress has been made in defining differences between visceral and subcutaneous adipose tissue, heterogeneity between individual subcutaneous depots remains comparatively underexplored despite its likely relevance to disorders of regional adipose expansion. The emergence of single-cell and spatial transcriptomic technologies has transformed the study of adipose tissue by enabling high-resolution mapping of adipocytes, stromal populations, vascular cells, and immune microenvironments within healthy and diseased tissue. These approaches offer an unprecedented opportunity to investigate depot-specific cellular states, intercellular signalling networks, and spatial tissue architecture in lipedema. In this review, we synthesise current evidence regarding tissue remodelling and adipose depot heterogeneity in lipedema and examine how single-cell and spatial omics approaches may advance mechanistic understanding of disease pathophysiology. We further discuss current technical and conceptual limitations within the field and highlight future directions for developing integrated adipose tissue atlases capable of identifying disease-driving cellular programs and therapeutic targets in lipedema.

## Lipedema as a depot-specific disease of adipose tissue

Lipedema is a chronic disorder of subcutaneous adipose tissue characterised by disproportionate fat accumulation predominantly affecting the lower extremities and, less commonly, the upper limbs, with relative sparing of the trunk. Clinically, the disease is associated with pain, tissue heaviness, easy bruising, oedema, and progressive impairment in mobility and quality of life. Lipedema occurs almost exclusively in women and frequently emerges during periods of hormonal transition, including puberty, pregnancy, and menopause (Foundation, 2022; Kruppa et al., 2023; Kruppa et al., 2026; Beltran and Herbst, 2017; Alwardat et al., 2019). Despite increasing recognition as a distinct clinical entity, lipedema remains substantially underdiagnosed and is frequently misclassified as obesity or lymphedema because of overlapping clinical features and the absence of validated molecular biomarkers (Foundation, 2022; Kruppa et al., 2026).

Although lipedema is defined by regional adipose expansion, its underlying biological basis remains incompletely understood. Historically, the disease has been viewed primarily as a disorder of abnormal fat deposition. However, emerging evidence suggests a more complex pathology involving coordinated remodelling of adipose tissue architecture, including adipocyte hypertrophy, dysregulation of adipose stem and progenitor cells (ASPCs), immune cell infiltration, vascular dysfunction, extracellular matrix (ECM) remodelling, and progressive lymphatic impairment (Kruppa et al., 2023; Ishaq et al., 2022; Stroh et al., 2022; von Atzigen et al., 2023; Felmerer et al., 2020a; Wolf et al., 2021). Many of these features overlap with obesity and other adipose disorders, raising a central unresolved question: why does pathology preferentially arise within specific subcutaneous depots, particularly those of the lower limbs?

Recent advances in adipose tissue biology provide a framework for addressing this question. Adipose depots are now recognised as anatomically and functionally distinct cellular ecosystems characterised by depot-specific developmental programs, stromal composition, vascular architecture, immune microenvironments, and adipogenic potential. Comparative studies of visceral and subcutaneous adipose tissue have demonstrated substantial heterogeneity in metabolic and inflammatory signalling, while emerging evidence indicates that similar heterogeneity also exists between individual subcutaneous depots (Vijay et al., 2020; Emont et al., 2022; Deutsch et al., 2020; Massier et al., 2023). These findings suggest that the regional susceptibility observed in lipedema may reflect intrinsic differences in adipose depot biology rather than uniform systemic dysregulation.

Understanding how depot-selective pathology develops requires approaches capable of resolving adipose tissue at both cellular and spatial resolution. Adipose tissue is highly heterogeneous, comprising mature adipocytes embedded within dynamic stromal, vascular, lymphatic, neural, and immune networks. Consequently, bulk transcriptomic analyses obscure important cell-type-specific changes by averaging signals across multiple populations. In contrast, single-cell and single-nucleus RNA sequencing, together with emerging spatial transcriptomic technologies, now enable construction of high-resolution adipose tissue atlases that define discrete cellular states and their spatial organisation in health and disease.

Within this framework, lipedema may be conceptualised not simply as abnormal adipose expansion, but as a disorder of spatially restricted adipose tissue remodelling characterised by coordinated alterations across stromal, immune, vascular, lymphatic, and adipocyte compartments within susceptible depots. However, the extent to which these changes reflect intrinsic vulnerability of lower limb adipose tissue versus regionally expressed systemic drivers remains unresolved. Addressing this distinction will require integrative multi-omic approaches capable of linking cellular identity with spatial and anatomical context across adipose depots.

In this review, we examine lipedema through the integrated frameworks of adipose depot heterogeneity and single-cell biology. We first summarise the major cellular and tissue-level alterations identified in lipedema adipose tissue, including changes in adipocytes, ASPCs, immune populations, vascular and lymphatic networks, and ECM remodelling. We then discuss how emerging adipose tissue atlases generated through single-cell and spatial transcriptomic technologies provide new insight into depot-specific adipose biology and may help explain the regional vulnerability characteristic of lipedema. Finally, we explore how integrating single-cell transcriptomics, spatial omics, and comparative depot analysis may advance mechanistic understanding of lipedema pathophysiology and guide future diagnostic and therapeutic strategies.

## Cellular consequences of adipose remodelling in lipedema

The pathological features of lipedema likely arise through coordinated alterations across multiple interacting cellular compartments within affected adipose depots. Rather than representing isolated defects within individual cell types, current evidence supports a model of disrupted tissue homeostasis involving adipocytes, adipose stem and progenitor cells (ASPCs), immune populations, vascular and lymphatic networks, and extracellular matrix remodelling. These interacting processes collectively shape the regional adipose microenvironment and may contribute to progressive tissue expansion, fibrosis, oedema, and impaired tissue function. To begin to understand the pathophysiology of lipedema, we must first highlight the cellular derangements within lipedema tissue architecture.

### The adipocyte–ASPC axis in regional adipose expansion

Adipocyte dysfunction and altered ASPC activity appear to represent closely interconnected components of adipose tissue remodelling in lipedema. Together, these populations regulate tissue expansion, adipocyte turnover, and structural adaptation within affected depots.

A consistent histological feature of lipedema is adipocyte hypertrophy within affected subcutaneous adipose tissue, particularly in the lower extremities, with several studies demonstrating progressive adipocyte enlargement across disease stages (Kruppa et al., 2023; Ishaq et al., 2022; von Atzigen et al., 2023; Felmerer et al., 2020a; Wolf et al., 2021). Importantly, within-subject comparisons between affected lower limb depots and relatively unaffected abdominal depots support the regional specificity of this phenotype, suggesting that adipocyte enlargement cannot be explained solely by systemic lipid excess (Kruppa et al., 2023; Ishaq et al., 2022). Although adipocyte hypertrophy is also observed in obesity, its persistence in both obese and non-obese individuals with lipedema indicates that local tissue context likely contributes substantially to this process (Al-Ghadban et al., 2019).

Rather than reflecting uniform expansion of a homogeneous adipocyte population, these changes may involve shifts in adipocyte transcriptional states and lipid-handling programs within the local microenvironment. Preliminary transcriptomic analyses suggest the presence of distinct adipocyte subpopulations in lipedema tissue, although these findings remain incompletely validated and lack spatial resolution (Pagani et al., 2024). Adipocyte hypertrophy may therefore represent a downstream manifestation of altered stromal signalling and progenitor behaviour rather than an exclusively adipocyte-autonomous defect.

ASPC populations provide a potential mechanistic link between local tissue signalling and adipocyte expansion. Early histological studies identified increased numbers of proliferating Ki67^+^CD34^+^ stromal cells within affected subcutaneous depots, localised predominantly to interstitial and perivascular regions consistent with adipose progenitor niches (Suga et al., 2009). These findings support the presence of enhanced stromal activity within lipedema tissue and are consistent with ongoing tissue remodelling.

Single-cell transcriptomic studies of human adipose tissue have further demonstrated that ASPCs comprise heterogeneous populations including multipotent stem-like cells, committed preadipocytes, and regulatory fibro-adipogenic progenitors with distinct transcriptional and functional properties (Vijay et al., 2020; Emont et al., 2022; Deutsch et al., 2020; Massier et al., 2023; Merrick et al., 2019; Schwalie et al., 2018). Within this framework, alterations in ASPC composition or activation state could directly influence adipocyte number, lipid storage capacity, and depot expansion. Although these hierarchical relationships are increasingly well characterised in healthy and obese adipose tissue, their specific organisation in lipedema remains poorly understood.

Importantly, ASPC activation and adipocyte hypertrophy are unlikely to represent independent sequential events. Instead, these processes probably interact dynamically through reciprocal signalling mechanisms. Altered progenitor activation may promote adipocyte expansion, while hypertrophic adipocytes may further reinforce stromal activation through mechanical stress, lipid signalling, and inflammatory mediators. This bidirectional interaction supports a model in which dysregulation of the adipocyte–ASPC axis contributes substantially to pathological adipose remodelling within affected depots.

However, whether these alterations reflect intrinsic programming of lower limb adipose progenitors or are secondary to systemic hormonal, inflammatory, or mechanical influences remains unresolved.

### Immune remodelling within the adipose microenvironment

Within the framework of adipose tissue remodelling, immune alterations in lipedema are best understood as context-dependent responses to changes in stromal, adipocyte, and vascular function rather than as primary inflammatory activation. Adipose tissue immune cells are tightly integrated within the tissue microenvironment and dynamically respond to changes in lipid flux, extracellular matrix structure, and vascular integrity.

Macrophage infiltration is a consistent feature of lipedema adipose tissue across multiple depots, including both lower limb and abdominal subcutaneous fat (Felmerer et al., 2020a; Al-Ghadban et al., 2019; Suga et al., 2009; Felmerer et al., 2020b; Cinti, 2005). However, current evidence does not support a uniform pro-inflammatory expansion. Instead, macrophage populations appear to undergo state-dependent redistribution within the tissue, reflecting functional adaptation to local remodelling processes.

Lipedema-associated macrophages are frequently enriched for CD163^+^ phenotypes, which are generally associated with tissue remodelling and resolution rather than classical inflammatory activation (Wolf et al., 2022). Functional studies further suggest bidirectional signalling between macrophages and adipose stromal cells, in which macrophage-derived mediators may promote lipid accumulation and stromal activation, thereby linking immune activity directly to adipose expansion.

Importantly, macrophage biology in adipose tissue is now understood as a continuum of transcriptional and functional states rather than discrete M1/M2 polarisation. Single-cell studies in adipose biology have identified multiple macrophage subsets, including perivascular, lipid-associated, and metabolically specialised populations, each occupying distinct spatial and functional niches. Within this framework, immune alterations in lipedema likely reflect redistribution and state transitions within existing macrophage programs rather than net immune expansion.

Chemokine signalling pathways, including the MIF–CD74 axis, have been implicated in macrophage recruitment and retention within lipedema tissue (Vasella et al., 2023), supporting active stromal–immune communication. In contrast, other immune populations such as T cells do not appear consistently altered across studies (Felmerer et al., 2020a), suggesting that adaptive immune activation is not a dominant feature of the disease. Similarly, mast cell numbers are not consistently increased (Al-Ghadban et al., 2019), although experimental modulation of mast cell activity may influence stromal proliferation and inflammatory signalling (Bonetti et al., 2023), indicating potential functional relevance despite limited quantitative evidence.

Overall, immune remodelling in lipedema is best interpreted as a secondary but functionally important component of tissue adaptation, amplifying stromal and adipocyte dysfunction within a constrained adipose microenvironment rather than initiating disease.

### Vascular-Lymphatic remodelling in lipedema

Vascular and lymphatic systems constitute an integrated functional network within adipose tissue that regulates nutrient delivery, interstitial fluid balance, and immune trafficking. In lipedema, these systems undergo coordinated but dysregulated remodelling in response to altered adipocyte, stromal, and immune signalling.

Endothelial dysfunction is a consistent feature of lipedema tissue and is characterised by structural and functional remodelling rather than simple vessel expansion. Histological analyses demonstrate increased capillary density, endothelial proliferation, and thickening of vascular walls within affected subcutaneous depots (Stroh et al., 2022; Al-Ghadban et al., 2019; Michelini et al., 2025; Allen et al., 2020; Al-Ghadban et al., 2023). In parallel, functional studies show that adipose-derived factors from lipedema tissue can disrupt endothelial barrier integrity and alter expression of junctional proteins including VE-cadherin, CD31, and ZO-1, suggesting increased vascular permeability (Stroh et al., 2022; Al-Ghadban et al., 2023).

The lymphatic system exhibits a more progressive pattern of impairment. Early disease stages do not consistently demonstrate reduced lymphatic vessel density (von Atzigen et al., 2023), whereas later stages are associated with lymphatic dilation and reduced drainage capacity. Molecular alterations in VEGF-C signalling and its receptors (VEGFR-3, Tie2) suggest dysregulated lymphangiogenic signalling rather than primary developmental defects (von Atzigen et al., 2023; Felmerer et al., 2020b). Functional lymphatic impairment is likely exacerbated by increased vascular leakage, ECM stiffening, and mechanical compression from expanding adipose tissue (Bilancini et al., 1995; Katzer et al., 2021).

Importantly, vascular and lymphatic dysfunction are tightly interdependent. Increased vascular permeability elevates interstitial fluid load, while impaired lymphatic clearance further amplifies tissue oedema and inflammatory signalling. This creates a reinforcing cycle of fluid accumulation and tissue remodelling that contributes to progressive disease severity.

Overall, vascular and lymphatic changes in lipedema are best interpreted as maladaptive responses to altered adipose tissue signalling rather than isolated primary defects.

### Extracellular vesicle–mediated communication

Extracellular vesicles (EVs), including exosomes and microvesicles, represent an additional layer of intercellular communication within adipose tissue and may contribute to coordinated tissue remodelling in lipedema. Adipose tissue is a major source of EV-associated microRNAs (miRNAs), which regulate gene expression in recipient cells through paracrine and autocrine signalling pathways (Thomou et al., 2017; Samanta et al., 2018).

In lipedema, altered EV-miRNA profiles have been associated with pathways involved in angiogenesis, lymphatic function, endothelial permeability, and inflammatory signalling (Priglinger et al., 2020). These findings suggest that EV-mediated communication may act as a systems-level amplifier of local tissue dysfunction, coordinating responses across adipocyte, stromal, immune, and vascular compartments.

More broadly, EVs provide a plausible mechanism for synchronising cellular states across spatially distinct regions of adipose depots. In this context, EV signalling may propagate local metabolic or inflammatory changes, contributing to the spatial expansion of pathological remodelling. Although evidence remains preliminary, EV-mediated communication represents a potentially important integrative mechanism linking cellular dysfunction to tissue-level coordination in lipedema.

### Extracellular matrix remodelling as a structural output of tissue dysfunction

Extracellular matrix (ECM) remodelling represents the structural and biomechanical manifestation of coordinated dysfunction across adipocyte, immune, and vascular compartments within the lipedema depot microenvironment. In healthy adipose tissue, ECM turnover maintains tissue compliance and permits dynamic expansion and contraction in response to metabolic demand. Disruption of this balance alters mechanical properties of the tissue and constrains cellular plasticity.

In lipedema, fibrosis and altered ECM organisation are frequently reported, with increased collagen deposition correlating with disease severity in several studies (Alwardat et al., 2019; Felmerer et al., 2020a). Macroscopically, affected tissue may display nodular fibrotic structures (“fat pearls”), suggesting spatially heterogeneous matrix accumulation within subcutaneous depots (Ishaq et al., 2022). These features are consistent with altered stromal activity and dysregulated matrix remodelling within the tissue ecosystem. However, ECM changes appear to be heterogeneous across anatomical layers and disease stages, with some studies reporting reduced collagen content in superficial biopsies (Chavakis et al., 2023). This variability likely reflects spatial differences in mechanical loading, vascular integrity, and inflammatory signalling within the depot, rather than a uniform fibrotic programme.

Mechanistically, ECM remodelling is tightly coupled to vascular permeability, immune cell activity, and adipocyte expansion. Excessive matrix deposition increases tissue stiffness, impairs vascular and lymphatic flow, and restricts adipocyte plasticity, thereby amplifying dysfunction across all compartments of the depot microenvironment (Rosen Evan and Spiegelman, 2014; Ghaben and Scherer, 2019). Conversely, altered ECM degradation may facilitate abnormal tissue expansion and interstitial fluid accumulation, reinforcing a self-perpetuating cycle of structural and cellular remodelling. Within this framework, ECM remodelling is best understood as the integrated mechanical outcome of depot-level ecosystem dysfunction rather than an isolated pathological endpoint.

**FIGURE 1:**
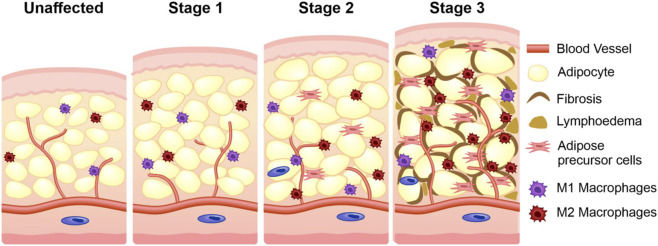
Changes in lipedema microenvironment tissue and cellular derangements in the lipedema microenvironment. From Stage 1 to Stage 3, progressive tissue remodelling include adipocyte hypertrophy, proliferative adipose stem and progenitor cells, infiltrating macrophages, interstitial fibrosis and lymphoedema.

Based on the collective evidence described above, the strongest emerging biology themes across lipedema include adipocyte dysfunction likely involving impaired differentiation and altered lipid handling; immune involvement, especially macrophage/myeloid alterations; stromal remodelling as evidence for altered ECM/fibrotic programs appears relatively consistent; vascular/lymphatic signaling abnormalities is plausible, but still not firmly established mechanistically. These progressive cellular and tissue-level alterations within the lipedema microenvironment support a model in which dysregulation of adipose stem and progenitor cells (ASPCs) drives the formation of hypertrophied, functionally compromised adipocytes. This process, likely modulated by hormonal influences, reinforces a self-perpetuating cycle of inflammation, vascular leakage, and fibrosis, ultimately contributing to impaired lymphatic drainage and progressive disease pathology (Figure 1).

## Adipose tissue atlases as a unifying framework for lipedema - depot heterogeneity, cellular drivers, and single-cell transcriptomic resolution

Resolving the question of how lipedema develops requires approaches capable of disentangling depot-intrinsic biology from disease-driven changes. A central limitation in lipedema research is the inability of bulk tissue analyses to resolve whether observed molecular signatures reflect true cellular dysfunction or shifts in cellular composition. Given the pronounced heterogeneity of adipose tissue, bulk transcriptomic approaches inevitably conflate signals from adipocytes, adipose stem and progenitor cells (ASPCs), immune cells, endothelial cells, and fibroblasts, obscuring the cellular basis of disease-associated changes. Understanding lipedema within a coherent biological framework requires integration of disease mechanisms with the emerging concept that adipose tissue is organised as a set of spatially and developmentally distinct cellular ecosystems. In this context, “fat atlases” generated through single-cell and spatial transcriptomic approaches provide a foundational reference for interpreting both normal depot heterogeneity and disease-associated deviations. These atlases move the field beyond descriptive histology, bulk cytokine profiling, and adipocyte-centric models, toward mechanistic frameworks that resolve cell–cell interactions and identify candidate driver populations underlying adipose dysfunction.

**FIGURE 2:**
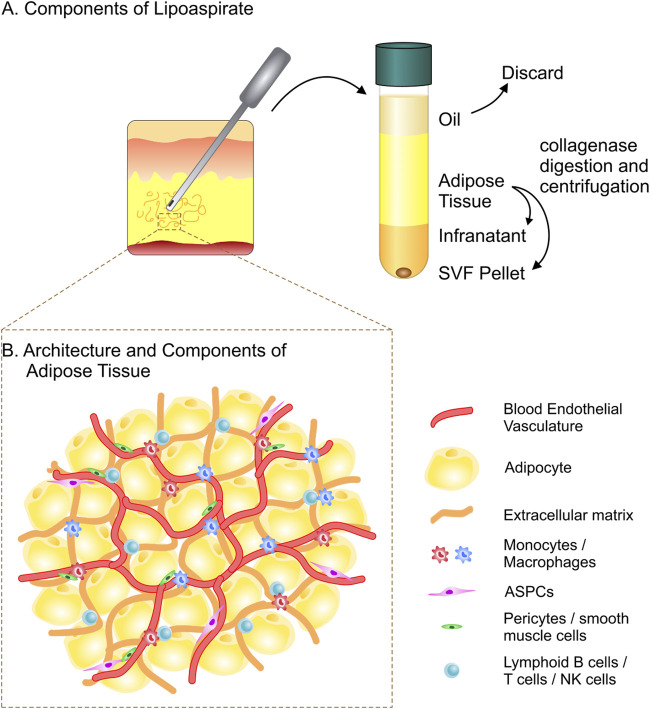
Microarchitecture of adipose tissue. **(A)** Schematic diagram depicting lipoaspiration of subcutaneous fat. Lipoaspirate is separated into layers of oil, adipose tissue, infranatant (blood, plasma and tumescent fluid) and a stromal vascular fraction (SVF) cell pellet after collagenase digestion and centrifugation. **(B)** The tissue architecture of adipose tissue and the cellular components of SVF. Adapted from Shukla et al. (2015).

Single-cell transcriptomics has revealed adipose tissue to be a structured but highly dynamic organ composed of interacting adipocytes, stromal vascular cells, immune populations, vascular networks, extracellular matrix (ECM) components, and neural inputs, revealing that adipose tissue is composed of dynamic and interrelated cellular states rather than fixed, homogeneous populations (Figure 2). By cell number, adipose tissue consists of approximately one-third mature adipocytes and two-thirds stromal vascular fraction (SVF). Adipocytes themselves are terminally differentiated, post-mitotic cells that occupy more than 90% of adipose tissue volume. Their primary functions include energy storage and endocrine signalling through adipokines such as leptin and adiponectin (Rosen Evan and Spiegelman, 2014; Wang et al., 2013; Skurk and Hauner, 2012). Lipid droplets are defined by a phospholipid monolayer surrounding triglyceride and sterol ester cores (Church et al., 2012). Single-nucleus RNA sequencing and spatial transcriptomics have further revealed that adipocytes are not a homogeneous population but instead comprise transcriptionally distinct subtypes likely reflecting developmental and environmental divergence (Emont et al., 2022; Bäckdahl et al., 2021). The SVF contains adipose stem and progenitor cells (ASPCs), vascular cells, and immune cells, previously defined through enzymatic digestion and flow cytometry approaches (Ishaq et al., 2022; Symonds, 2012; Zuk et al., 2002). Single-cell transcriptomics has since refined this compartment into discrete cellular hierarchies, revealing functional subpopulations with distinct transcriptional and developmental signatures (Maniyadath et al., 2023).

This framework is particularly relevant to disorders of regional adipose expansion such as lipedema, where pathology is anatomically restricted rather than systemic. In this context, fat atlases are not merely descriptive tools but essential reference maps for identifying deviations from homeostatic cellular states.

### Depot heterogeneity as a central biological principle

**FIGURE 3:**
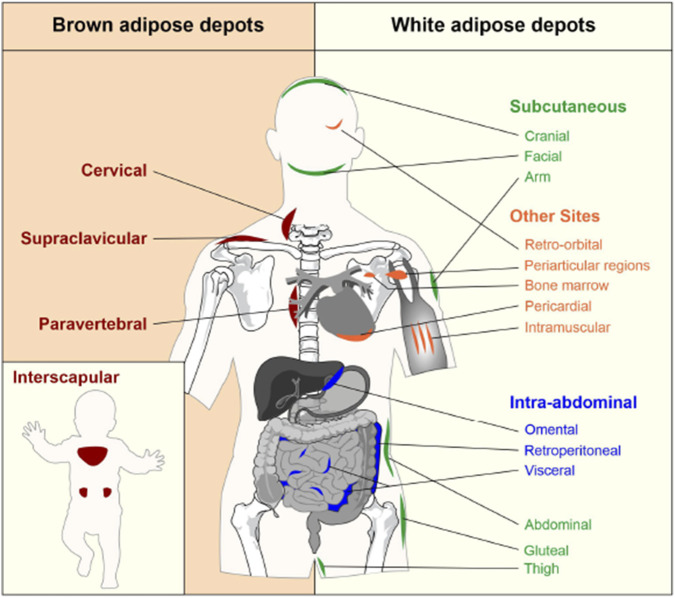
Adipose depots. In humans, adipose tissue is distributed throughout the body, with subcutaneous adipose and intra-abdominal adipose representing the main adipose storage depots. Brown adipose tissue, for non-shivering thermogenesis, is profuse at birth. Although it dissipates with age, some brown adipose tissue remains in adulthood. Figure adapted from Gesta et al. (2007).

Adipose tissue is found in locations throughout the body and may be sub-divided into different sub-types based on the storage location or “depot” (Figure 3). It may be divided into either white adipose tissue (WAT) or brown adipose tissue (BAT), which are anatomically, morphologically, and functionally distinct. The two major subcategories of WAT display distinct metabolic behaviour and are divided anatomically into visceral adipose tissue (VAT) and subcutaneous adipose tissue (SAT) “niches” A recurring theme across adipose biology is that depot identity is encoded at multiple levels: developmental lineage, transcriptional programming, stromal composition, vascular architecture, and immune niche organisation. Differences between visceral and subcutaneous adipose tissue demonstrate that these depots are not interchangeable but instead represent distinct biological systems or “mini organs” with unique functional constraints (Chau et al., 2014; Tchkonia et al., 2007; Gesta et al., 2006.; Tchkonia et al., 2005; Tchkonia et al., 2006; van Harmelen et al., 2004; Tchkonia et al., 2002; Jensen et al., 2003; Jensen, 1995; Wiest et al., 2010; Kovacova et al., 2012; Fontana et al., 2007; Cartier et al., 2008). For example, depot-specific expression of developmental regulators such as HOX genes, TBX15, SHOX2, and EN1 suggests that positional identity is partially maintained into adulthood (Tchkonia et al., 2007; Gesta et al., 2006.; Pinnick et al., 2014; Karastergiou et al., 2013; Gehrke et al., 2013). These differences persist in cultured cells, indicating intrinsic programming rather than purely environmental influence. Similarly, differences in lipid storage capacity, lipolytic responsiveness, and adipokine secretion profiles further support the notion that adipose depots are functionally specialised (Jensen et al., 2003; Jensen, 1995; Wiest et al., 2010; Kovacova et al., 2012; Fontana et al., 2007; Cartier et al., 2008).

While visceral versus subcutaneous comparisons have been extensively studied, far less is known about heterogeneity within subcutaneous adipose tissue itself. This represents a critical gap in current fat atlas construction. Subcutaneous depots located in the abdomen, gluteofemoral region, thighs, arms, and lower limbs exhibit distinct metabolic and structural properties (Pinnick et al., 2014; Karastergiou et al., 2013; Gehrke et al., 2013; Karpe and Pinnick, 2015; Fraser et al., 2007; Hauner and Entenmann, 1991; Tchoukalova et al., 2010; Gao et al., 2017; Li et al., 2019; Al-Sari et al., 2020; Pinnick et al., 2012; Lafontan et al., 1979; Wahrenberg et al., 1989). However, these depots are often treated as a single entity in transcriptomic analyses, despite evidence that they differ in adipocyte size and lipid composition, progenitor proliferation rates, adipogenic differentiation potential, lipolytic responsiveness, adipokine and lipokine secretion profiles (Gehrke et al., 2013; Fraser et al., 2007; Hauner and Entenmann, 1991; Tchoukalova et al., 2010; Gao et al., 2017; Li et al., 2019; Al-Sari et al., 2020; Pinnick et al., 2012; Lafontan et al., 1979; Wahrenberg et al., 1989; Cao et al., 2008). Further, single cell transcriptomic analyses of VAT and SAT tissue have consistently demonstrated that VAT-enriched specific ASPC clusters express mesothelin, a marker of mesothelial cells, whilst the SAT-enriched clusters do not (Vijay et al., 2020). In addition to differences in molecular markers between VAT and SAT, functional comparisons have also shown further differences in cellular proliferation, adipogenesis, apoptosis, fatty-acid storage, lipolysis and adipokine secretion. For example, subcutaneous and mesenteric preadipocytes proliferated more rapidly than omental counterparts (Tchkonia et al., 2005; Tchkonia et al., 2006; van Harmelen et al., 2004). Similarly, in several studies, subcutaneous and visceral preadipocytes appeared to have different abilities to undergo adipogenic differentiation (Tchkonia et al., 2005; Tchkonia et al., 2006; Tchkonia et al., 2002), whereas other studies showed no difference in adipogenic capacity (van Harmelen et al., 2004). Developmental gene expression differences between upper and lower body subcutaneous depots further suggest that these regions are biologically distinct rather than variations of a single tissue type (Pinnick et al., 2014; Karastergiou et al., 2013; Gehrke et al., 2013). However, unlike visceral versus subcutaneous comparisons, there are currently no comprehensive single-cell atlases that resolve these differences at cellular resolution. This missing layer of spatial and anatomical resolution within and across adipose depots is particularly relevant to lipedema, which preferentially affects specific subcutaneous depots while sparing others. The absence of a complete subcutaneous fat atlas therefore represents a fundamental limitation in current disease modelling.

### Cellular drivers of adipose expansion: integrating stromal, immune, and vascular signals with cell-cell communication

Within this framework, scRNA-seq provides a particularly powerful lens for interrogating lipedema because it allows disease-associated changes to be mapped onto specific cellular compartments within the adipose ecosystem. Rather than treating adipose tissue as a uniform structure undergoing global expansion, single-cell approaches enable identification of which cell populations are expanded, which are transcriptionally reprogrammed, and how intercellular signalling networks are altered within affected depots.

**FIGURE 4:**
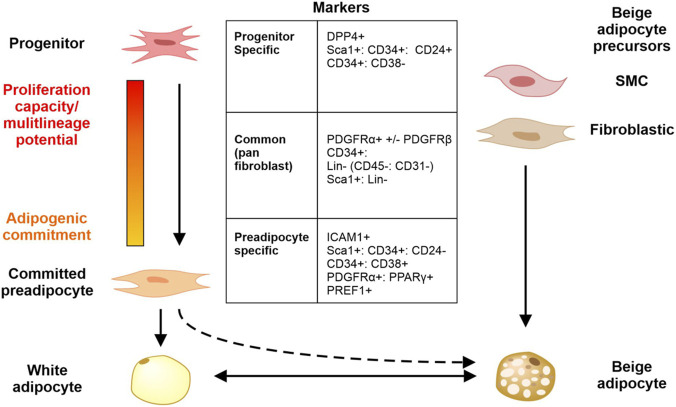
Adipose stem and progenitor hierarchy. Adipocyte precursor populations exist along a spectrum of differentiation, reflecting varying levels of commitment to the adipogenic lineage. Current evidence indicates that adipocytes predominantly arise from perivascular fibroblastic populations, which are characterised by shared fibroblastic markers such as *Pdgfra* and *Cd34*, and are notably devoid of hematopoietic or endothelial markers (i.e., CD45-:CD31-, abbreviated “Lin”). These progenitor cells give rise to both white and beige adipocytes, with the capacity for phenotypic switching in response to environmental temperature. ACTA2: Actin Alpha 2. CTGF: Connective Tissue Growth Factor. DPP4: Dipeptidyl Peptidase-4. ICAM1: Intercellular Adhesion Molecule 1. Ly6C: Lymphocyte Antigen 6C. PDGFAA: Platelet Derived Growth Factor AA. PDGFRa/b: Platelet Derived Growth Factor Receptor Alpha/Beta. PREF: Preadipocyte Factor 1. Sca-1: Stem Cell antigen-1. Adapted from Sakers et al. (2022).

In adipocyte and ASPC compartments, single-cell studies have the potential to resolve whether adipocyte hypertrophy in lipedema reflects intrinsic shifts in lipid-handling programs, altered progenitor differentiation trajectories, or expansion of specific adipocyte sub-states with distinct metabolic phenotypes. Single-cell transcriptomic studies have allowed the sequencing of human subcutaneous adipose tissue and the identification of a hierarchy of ASPC sub-populations within it. Similarly, ASPCs can be subdivided into multipotent stem-like populations, committed preadipocytes, and regulatory fibro-adipogenic progenitors with distinct transcriptional and functional properties (Figure 4) (Emont et al., 2022; Massier et al., 2023; Merrick et al., 2019; Schwalie et al., 2018). scRNA-seq enables interrogation of whether lipedema is associated with expansion of proliferative progenitors, bias toward adipogenic commitment, or dysregulation of regulatory stromal populations that normally constrain adipose expansion. This distinction is critical, as it allows reinterpretation of ASPC “activation” in lipedema as either a compensatory repair response or a primary driver of pathological tissue remodelling.

**FIGURE 5:**
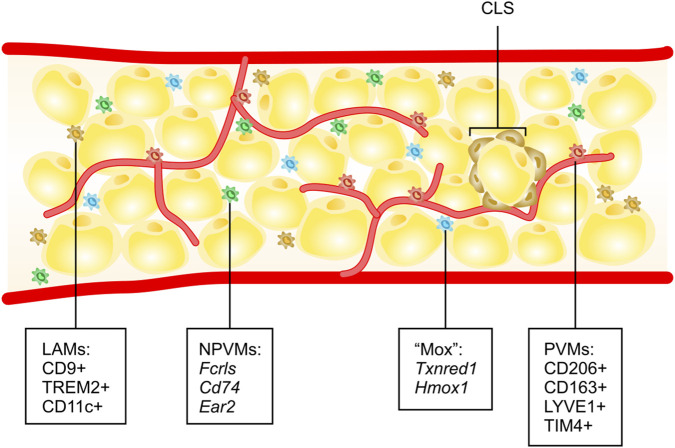
Proposed adipose tissue macrophage sub-types. Resident adipose tissue macrophages are perivascular macrophages (PVMs) or not associated with blood vessels (NPVMs). PVMs are positive for CD206, LYVE1, CD163 and TIM4, whereas NPVMs have high expression of Fcrls, Cd74 and Ear2. Lipid associated macrophages (LAMs) are marked by CD9, CD11c and TREM2. They may gather in crownlike structure (CLS) around damaged or dying adipocytes. “Mox” macrophages have high expression of Txnred1 and Hmox1. Adapted from Chavakis et al. (2023).

In immune compartments, single-cell approaches move beyond binary M1/M2 paradigms by revealing macrophage heterogeneity across perivascular, lipid-associated, and tissue-remodelling states, as well as identifying rare or transient immune populations that may be missed in bulk analyses and therefore has become apparent that the M1-M2 dichotomy is overly simplistic (Figure 5) (Chavakis et al., 2023). This is particularly relevant in lipedema, where macrophage infiltration is a consistent finding (Felmerer et al., 2020a; Al-Ghadban et al., 2019; Suga et al., 2009; Cinti, 2005), scRNA-seq provides a means to determine whether immune involvement reflects true expansion of inflammatory populations or redistribution of macrophage functional states in response to altered lipid handling, vascular stress, or stromal remodelling.

Importantly, this framework also enables re-evaluation of lymphoid compartments. While T cell abundance does not appear consistently altered in bulk analyses (Suga et al., 2009), single-cell resolution is required to determine whether subtle shifts in T cell activation state, tissue residency, or spatial localisation contribute to disease biology. Single-cell profiling further enables evaluation of lymphoid compartments (T cells, B cells, NK cells) at sufficient resolution to determine whether adaptive immune involvement is truly minimal or instead characterised by subtle shifts in state or localisation (Emont et al., 2022; Massier et al., 2023; Hildreth et al., 2021; Srikakulapu and McNamara, 2020).

In vascular and lymphatic compartments, scRNA-seq allows endothelial and mural cells to be subdivided into functionally distinct transcriptional states, including angiogenic, inflammatory, barrier-regulating, and lymphatic endothelial subtypes. This is particularly important for lipedema, where endothelial dysfunction and impaired fluid handling are central but mechanistically unresolved features (Stroh et al., 2022; Al-Ghadban et al., 2019; Allen et al., 2020; Al-Ghadban et al., 2023). Single-cell resolution enables assessment of whether these changes reflect endothelial activation, phenotypic switching, or expansion of specific vascular subpopulations within affected depots.

A key advantage of single-cell approaches is their ability to reconstruct intercellular communication networks through ligand–receptor inference. This allows identification of signalling axes linking adipocytes and ASPs that regulate adipose expansion through proliferation and differentiation into adipocytes (Vijay et al., 2020; Emont et al., 2022; Merrick et al., 2019), immune cells that modulate inflammation, remodelling, and adipocyte turnover (Chavakis et al., 2023; Hildreth et al., 2021; Srikakulapu and McNamara, 2020; Suganami and Ogawa, 2010; Weisberg et al., 2003; Gordon and Taylor, 2005; Mills, 2015; Wang et al., 2019; Allah et al., 2017; Silva et al., 2019; Chakarov et al., 2019; Serbulea et al., 2018.; Jaitin et al., 2019), endothelial cells that regulate nutrient delivery, angiogenesis, and oxygen availability (Eelen et al., 2018; Kalucka et al., 2018; Shayan et al., 2008), and ECM and stromal cells (fibroblasts), that regulate mechanical constraints and differentiation niches (Vijay et al., 2020; Emont et al., 2022; Deutsch et al., 2020; Massier et al., 2023; Merrick et al., 2019) providing a systems-level view of how tissue remodelling is coordinated. In lipedema, such analyses are particularly relevant for identifying how stromal–immune interactions regulate adipose expansion, how endothelial signalling responds to adipocyte-derived cues, and how immune populations contribute to vascular permeability and extracellular matrix remodelling. In lipedema, dysregulated expansion likely reflects perturbations across all four axes rather than a single initiating cell type. However, current evidence does not allow prioritisation of any one compartment as the primary driver. Importantly, cell–cell communication networks inferred from scRNA-seq data suggest that adipose tissue function emerges from feedback loops rather than linear hierarchies. For example, endothelial–ASPC signalling regulates adipogenesis and vascular expansion; macrophage–adipocyte interactions regulate lipid handling and inflammation and ECM stiffness influences progenitor differentiation trajectories.

This framework shifts the interpretation of lipedema from isolated cellular abnormalities to coordinated dysfunction of interacting cellular networks within a spatially constrained tissue ecosystem.

### Single-cell transcriptomics as a mechanistic lens for lipedema biology

Understanding lipedema as a disorder of regional adipose expansion requires cellular and molecular resolution of the affected tissue. This limitation is particularly relevant in lipedema, where histological and molecular changes occur within spatially restricted depots and are likely driven by coordinated shifts across several interacting cellular populations.

Single-cell RNA sequencing (scRNA-seq) and single-nucleus RNA sequencing (snRNA-seq) have therefore emerged as transformative tools for adipose tissue biology, enabling the deconvolution of tissue complexity into discrete cellular states and lineage trajectories (Vijay et al., 2020; Emont et al., 2022; Deutsch et al., 2020; Massier et al., 2023; Merrick et al., 2019). These approaches have already reshaped understanding of adipose tissue in health and obesity by revealing previously unrecognised heterogeneity among adipocytes, adipose stem and progenitor cells (ASPCs), endothelial populations, fibroblasts, and immune subsets. Importantly, they have demonstrated that many classical “cell types” in adipose tissue are better understood as dynamic cellular states embedded within continuous transcriptional landscapes.

However, scRNA-seq alone is insufficient to fully resolve lipedema biology due to the loss of spatial context during tissue dissociation. Adipose tissue is highly structured, with functional niches organised around vasculature, fibrotic septa, and lymphatic networks. Consequently, disease-relevant interactions may be spatially restricted and therefore obscured in dissociated single-cell datasets. This limitation is particularly important in lipedema, where pathology appears to follow depot-specific and regionally patterned distribution within the lower extremities.

For this reason, integration of single-cell atlases with spatial transcriptomics and imaging-based modalities is essential. Spatially resolved approaches preserve tissue architecture, enabling mapping of transcriptional states onto anatomical structures and identification of microenvironmental niches where disease processes are concentrated. In lipedema, this would allow direct interrogation of whether immune activation, vascular dysfunction, and fibrotic remodelling occur diffusely or are localised to specific stromal or vascular domains within the depot.

Taken together, single-cell transcriptomics provides a foundational methodological framework for redefining lipedema as a disorder of coordinated cellular state transitions within a spatially restricted adipose ecosystem. By resolving tissue complexity into defined cellular programs and interaction networks, scRNA-seq enables a shift from descriptive pathology toward mechanistic understanding. When integrated with spatial and depot-resolved adipose atlases, this approach offers a pathway to determine whether lipedema arises from aberrant activation of conserved adipose programs, failure of depot-specific regulatory networks, or a combination of both operating within vulnerable lower limb adipose tissue. The integration of spatial transcriptomics with single-cell atlases therefore represents a key methodological advance for resolving these limitations.

Collectively, adipose tissue atlases provide both a conceptual and technical framework for bridging major knowledge gaps in lipedema research. By embedding lipedema within a structured map of depot heterogeneity, it becomes possible to reinterpret disease features not as isolated pathological findings, but as alterations in defined cellular programs operating within specific anatomical contexts. This atlas-based perspective is essential for determining whether lipedema represents a systemic adipose dysregulation with regional expression, or a fundamentally depot-intrinsic disorder arising from unique lower limb adipose ecosystem architecture.

## Depot-specific responsiveness: a missing mechanistic dimension in lipedema

One of the most important implications arising from adipose tissue heterogeneity obtained from ‘fat atlases’ is that adipose depots are unlikely to respond uniformly to identical physiological, hormonal, mechanical, or inflammatory stimuli. Rather than functioning as passive lipid storage compartments, adipose depots appear to represent regionally specialised cellular ecosystems with intrinsic developmental programming, distinct stromal composition, and unique immune–vascular architecture. This concept has major implications for understanding lipedema yet remains poorly explored experimentally.

Evidence from obesity and metabolic disease research already demonstrates that adipose depots exhibit markedly different biological responses under equivalent systemic conditions. Visceral and subcutaneous adipose tissues differ substantially in insulin sensitivity, fatty acid handling, and lipolytic responsiveness (Jensen et al., 2003; Jensen, 1995). Visceral adipose tissue demonstrates greater catecholamine-induced lipolysis and relative resistance to insulin-mediated suppression of lipid mobilisation compared with subcutaneous adipose tissue, contributing to distinct metabolic outcomes (Jensen et al., 2003; Jensen, 1995). Similarly, depot-specific differences in adipokine secretion have been repeatedly demonstrated, with subcutaneous depots generally producing higher levels of leptin, whereas visceral depots display greater expression of pro-inflammatory mediators including IL-6 and TNF-α (Wiest et al., 2010; Kovacova et al., 2012; Fontana et al., 2007; Cartier et al., 2008). These findings suggest that adipose depots possess fundamentally distinct endocrine and inflammatory set-points.

Importantly, these functional differences are increasingly understood to arise from differences in underlying cellular composition and developmental programming. Single-cell transcriptomic analyses have demonstrated that adipose stem and progenitor cell (ASPC) populations differ substantially between depots, both in abundance and transcriptional identity (Vijay et al., 2020; Emont et al., 2022; Massier et al., 2023). Visceral depots contain mesothelial-like progenitor populations expressing WT1 and mesothelin-associated programs that are largely absent in subcutaneous adipose tissue (Vijay et al., 2020; Chau et al., 2014). Conversely, subcutaneous depots demonstrate enrichment of developmental patterning genes including TBX15, SHOX2, EN1 and HOXC9 (Tchkonia et al., 2007; Gesta et al., 2006). These depot-specific progenitor programs may contribute to regional differences in adipogenic capacity, tissue expansion, and ECM remodelling potential.

Functional studies further support this concept. Adipogenic differentiation capacity varies between depots, although results remain somewhat inconsistent depending on methodology and anatomical site studied (Tchkonia et al., 2005; Tchkonia et al., 2006; van Harmelen et al., 2004; Tchkonia et al., 2002; Gehrke et al., 2013; Fraser et al., 2007; Hauner and Entenmann, 1991; Tchoukalova et al., 2010; Gao et al., 2017; Li et al., 2019). Some studies demonstrate greater proliferative and adipogenic capacity within femoral and gluteofemoral progenitors compared with abdominal depots (Fraser et al., 2007; Gao et al., 2017; Li et al., 2019), whereas others report enhanced adipogenesis in abdominal SAT (Hauner and Entenmann, 1991; Tchoukalova et al., 2010). Despite these inconsistencies, the broader theme remains clear: progenitor behaviour is not uniform across adipose depots. Instead, local progenitor populations appear to possess intrinsic depot-specific biases that influence tissue expansion, lipid accumulation, and remodelling responses.

Depot-specific immune responsiveness represents another emerging layer of adipose heterogeneity. Single-cell studies have shown substantial variation in immune cell composition between depots, including differences in macrophage populations, lymphoid subsets, and endothelial-associated immune niches (Emont et al., 2022; Massier et al., 2023; Hildreth et al., 2021). Visceral adipose tissue generally demonstrates greater inflammatory cell enrichment under metabolic stress conditions, whereas subcutaneous depots exhibit relatively distinct immune architectures (Emont et al., 2022; Massier et al., 2023). Importantly, macrophage populations themselves are highly heterogeneous, encompassing homeostatic perivascular macrophages, inflammatory lipid-associated macrophages (LAMs), oxidative stress-responsive Mox macrophages, and multiple transitional states (Chavakis et al., 2023; Silva et al., 2019; Chakarov et al., 2019; Serbulea et al., 2018.; Jaitin et al., 2019). The proportional representation and activation state of these populations likely differs regionally between depots, potentially influencing local tissue remodelling, fibrosis, angiogenesis, and adipocyte turnover.

These observations collectively support the concept that adipose depots are effectively “pre-programmed” to interpret systemic cues differently. In this framework, developmental origin, stromal composition, vascular architecture, extracellular matrix organisation, and immune niche composition all shape how a given depot responds to hormonal, metabolic, or inflammatory stimuli. Thus, identical circulating signals may generate profoundly different tissue-level outcomes depending on depot identity.

This concept may be particularly important in lipedema. A central unresolved question in lipedema biology is why disease selectively affects specific subcutaneous depots despite exposure of the entire body to the same systemic endocrine environment. The striking regional distribution of lipedema tissue strongly suggests that local tissue susceptibility contributes substantially to disease pathogenesis. In this context, lipedema may not simply arise from abnormal signalling alone, but rather from altered interpretation of physiological signals by depot-specific cellular ecosystems. Under such a model, gluteofemoral and lower extremity depots affected by lipedema may possess intrinsic stromal, vascular, immune, or progenitor programs that predispose them to maladaptive tissue remodelling when exposed to otherwise normal hormonal or inflammatory stimuli. Depot-specific ASPC populations may exhibit exaggerated proliferative responses, altered adipogenic differentiation trajectories, or aberrant extracellular matrix production. Similarly, depot-specific endothelial or lymphatic populations may display differential susceptibility to vascular leak, hypoxia, or impaired interstitial fluid handling. Local immune populations may also respond differently to tissue stress, generating chronic low-grade inflammatory or fibrotic signalling states that perpetuate regional tissue expansion.

Importantly, current lipedema studies remain insufficient to test these hypotheses rigorously. Most existing transcriptomic analyses compare lipedema tissue to control tissue without adequately accounting for anatomical depot identity. This represents a major limitation because depot-specific biological differences may confound interpretation of disease-associated signatures. Without anatomically matched adipose atlases, it becomes difficult to distinguish true disease-specific transcriptional programs from normal regional adipose variation.

## Towards a convergent systems model of lipedema

Emerging adipose atlas data, depot biology studies, and preliminary lipedema transcriptomic analyses support a convergent systems model of lipedema pathophysiology. Rather than arising from a single pathological mechanism, lipedema likely reflects the interaction of multiple biologically permissive layers operating within anatomically susceptible adipose depots. This framework may help explain why lipedema selectively affects particular regions, despite uniform systemic hormonal and metabolic exposure.

At the foundation of this model is depot-specific susceptibility. Adipose depots possess distinct developmental origins, transcriptional programs, vascular architecture, immune niches, and stromal composition (Vijay et al., 2020; Emont et al., 2022; Massier et al., 2023; Chau et al., 2014; Tchkonia et al., 2007; Gesta et al., 2006). These intrinsic differences likely establish varying capacities for adipose expansion, inflammation, fibrosis, and vascular remodelling. The characteristic lower extremity distribution of lipedema therefore suggests that certain subcutaneous depots may be inherently predisposed to maladaptive tissue responses.

A second layer involves cellular composition of the adipose microenvironment. Single-cell studies demonstrate that adipose tissue is composed of diverse populations of adipocytes, adipose stem and progenitor cells (ASPCs), immune cells, endothelial cells, lymphatic cells, and stromal support cells (Vijay et al., 2020; Emont et al., 2022; Deutsch et al., 2020; Massier et al., 2023; Merrick et al., 2019). Importantly, the abundance and transcriptional states of these populations vary between depots. In lipedema, altered ASPC behaviour, adipocyte state transitions, macrophage activation, and stromal–vascular interactions may collectively drive progressive tissue expansion and remodelling.

A third component is the microenvironmental layer, incorporating extracellular matrix (ECM) organisation, vascular integrity, and lymphatic function. Adipose expansion requires coordinated angiogenesis, ECM remodelling, immune regulation, and interstitial fluid handling (Gupta Rana et al., 2012; Tang et al., 2008; Han et al., 2011; Cho et al., 2007). Dysfunction within these systems may promote hypoxia, capillary fragility, fibrosis, oedema, and chronic inflammatory signalling. Importantly, baseline differences in ECM composition and vascular–lymphatic architecture between depots may influence regional susceptibility to disease progression.

Finally, adipose depots exhibit differential responsiveness to systemic signals, including hormones, inflammatory mediators, catecholamines, and metabolic stressors (Jensen et al., 2003; Jensen, 1995; Wiest et al., 2010; Kovacova et al., 2012; Fontana et al., 2007; Cartier et al., 2008), (Gehrke et al., 2013; Fraser et al., 2007; Hauner and Entenmann, 1991; Tchoukalova et al., 2010; Gao et al., 2017; Li et al., 2019; Al-Sari et al., 2020; Pinnick et al., 2012; Lafontan et al., 1979; Wahrenberg et al., 1989; Cao et al., 2008). Consequently, identical systemic cues may generate distinct tissue-level responses depending on local depot identity. This concept may help explain why lipedema frequently emerges during hormonal transitions such as puberty, pregnancy, and menopause.

Together, these findings support a model in which lipedema reflects dysregulated regional adipose biology rather than a uniform systemic adipose disorder. Depot identity, cellular composition, microenvironmental regulation, and signalling responsiveness likely interact dynamically to drive selective adipose expansion and tissue remodelling. This systems-level framework highlights the importance of anatomically resolved adipose atlases and integrated single-cell approaches to distinguish true disease-specific mechanisms from normal regional adipose heterogeneity.

## Future directions and outstanding questions

Recent advances in single-cell and spatial transcriptomic technologies have transformed understanding of adipose tissue biology and provide an important framework for future lipedema research. However, despite increasing recognition that lipedema represents a biologically distinct disorder of regional adipose remodelling as outlined in the recent consensus-based position paper from the Lipedema World Alliance (Kruppa et al., 2026), many fundamental mechanistic questions remain unresolved. Current studies remain limited by small sample sizes, inconsistent anatomical sampling, and inadequate consideration of normal depot-specific adipose heterogeneity. Consequently, it is often difficult to distinguish true disease-associated transcriptional programs from baseline regional adipose variation. Future research will therefore require the development of comprehensive, anatomically resolved adipose “fat atlases” integrating single-cell transcriptomics, spatial biology, immune profiling, vascular and lymphatic mapping, and extracellular matrix analysis across multiple adipose depots. These approaches may help determine whether lipedema contains truly disease-specific cellular states or instead reflects dysregulated activation of normal depot-specific biological programs.

Several major outstanding questions remain. It is still unclear why lipedema selectively targets specific subcutaneous depots despite uniform systemic hormonal exposure. Similarly, the relative contributions of adipose stem and progenitor cell (ASPC) dysfunction, immune activation, vascular fragility, lymphatic impairment, and extracellular matrix remodelling to disease initiation remain uncertain. Whether inflammatory, vascular, and fibrotic signatures represent primary pathogenic drivers or secondary adaptive responses also remains unresolved. In addition, the extent to which developmental programming and depot-specific transcriptional identity influence disease susceptibility is largely unknown.

Future studies should therefore focus on understanding how cellular composition, stromal architecture, vascular–lymphatic networks, and extracellular matrix organisation differ between anatomically distinct depots and how these systems interact during disease progression. Integrating depot biology with lipedema transcriptomic data may clarify why affected tissues display selective expansion, altered mechanical properties, resistance to weight loss, and variable progression between individuals. Importantly, future lipedema research will likely require a shift away from reductionist models focused solely on adipocyte hypertrophy, inflammation, or lymphatic dysfunction in isolation. Instead, systems-level approaches recognising adipose tissue as a dynamic and regionally specialised organ composed of interacting cellular ecosystems may provide a more accurate framework for understanding disease biology.

Ultimately, integrating fat atlas technologies with depot-specific adipose biology has the potential to redefine lipedema pathophysiology. Such approaches may not only improve mechanistic understanding of disease initiation and progression, but also facilitate the development of biologically targeted diagnostic tools and therapies tailored to the unique regional biology of affected adipose tissue.
